# Hypoxia-Induced TGFBI Promotes Bladder Cancer Progression by Creating a Stemness Regulation Loop through Stabilizing the Disulfide Bonds of GDF15

**DOI:** 10.34133/research.1134

**Published:** 2026-02-11

**Authors:** Gaojie Zhang, Linfeng Wang, Guozhi Zhao, Yong Huang, Jiang Yu, Yang Cao, Rui Sun, Qiuchen Li, Ziling Wei, Yu Jiang, Yueqiang Peng, Weiyang He, Yongpeng Xie, Jiayu Liu

**Affiliations:** ^1^ Department of Urology, First Affiliated Hospital of Chongqing Medical University, Chongqing, China.; ^2^ School of Clinical Medicine, Chongqing Medical University, Chongqing, China.; ^3^ Department of Urology, First Hospital of Jilin University, Changchun, China.; ^4^ Department of Urology, Peking Union Medical College, Beijing, China.

## Abstract

The maintenance and regulation of cancer stem cell (CSC) stemness are crucial for tumor progression; however, the mechanisms underlying tumor stemness regulation remain poorly understood. Herein, we discovered that the enhanced hypoxia-induced transforming growth factor beta induced protein (TGFBI) in bladder cancer (BLCA) promotes the establishment of a stemness loop in the tumor microenvironment, facilitating the maintenance of CSC stemness and malignant proliferation. Clinically, the upregulation of hypoxic TGFBI in BLCA correlates with malignant BLCA features and poor prognosis. Mechanically, TGFBI can stabilize the structural integrity of disulfide bonds in Cys48 and Cys77 of growth differentiation factor 15 (GDF15), leading to aberrant function activity of GDF15 and secretion. Interestingly, secreted GDF15 consequently not only further upregulates CSC-related gene expression but also induces the activation of cancer-associated fibroblasts through the transforming growth factor beta receptor type 2 (TGFBR2)–transforming growth factor β (TGFβ)–TGFBI self-regulatory feedback loop to promote stemness in BLCA. TGFBI knockdown or GDF15 inhibition results in a decrease in functional proteins associated with stemness maintenance, which suppresses bladder CSCs’ self-renewal and effectively improves the efficacy of chemotherapy. Together, these findings demonstrate the pivotal role of TGFBI in BLCA’s stemness maintenance and BLCA progression, highlighting that the inhibition of the TGFBI/GDF15 axis is a potential therapeutic strategy for the amelioration of cancer chemotherapy.

## Introduction

Bladder cancer (BLCA) is among the most prevalent malignancies worldwide, with roughly 573,000 new cases and around 213,000 deaths recorded each year [1]. Roughly 75% of patients are diagnosed with non-muscle-invasive bladder cancer (NMIBC), whereas the rest are identified as having muscle-invasive bladder cancer (MIBC). Although therapeutic interventions are actively applied, the prognosis for patients with MIBC is still unsatisfactory, with over half of these patients developing metastatic disease within 3 years [2]. Furthermore, nearly 10% to 30% of NMIBC cases eventually progress to MIBC. In addition, about half of newly diagnosed patients, as well as those with metastatic disease, experience recurrence, which frequently results in distant metastasis and poor prognoses [3,4]. Consequently, elucidating the molecular and biological mechanisms that drive BLCA progression and metastasis has become an urgent priority.

Cancer stem cells (CSCs), frequently recognized as initiating cells of tumor, represent the most aggressive fraction of malignant cells and participate in multiple stages of tumor progression, such as initiation, relapse, and metastasis. Evidence has demonstrated that bladder CSCs not only are present but are also critically influenced by aberrancies in transforming growth factor β (TGFβ) signaling, which promotes oncogenic transformation and malignant proliferation [5–7]. Moreover, the maintenance of BLCA stemness is dependent not only on intrinsic molecular modifications but also on external signals derived from the tumor microenvironment (TME), including the hypoxic microenvironment and cancer-associated fibroblasts (CAFs) [8–10]. While tumor progression is affected by the combined effects of intrinsic cellular factors and the TME, it is still poorly understood how CSCs confer self-renewal and tumorigenic capacities in BLCA.

The transforming growth factor beta induced protein (TGFBI) serves as a component of the extracellular matrix (ECM) characterized by 4 fasciclin-1 areas and an arginine-glycine-aspartic (RGD) motif located at its C-terminal region [11,12], which participates in diverse biological events that contribute to tumor development and progression [11–14]. The upregulation of TGFBI has been previously observed in multiple malignancies, including breast, colorectal, prostate, glioma, osteosarcoma, hepatocellular, and ovarian cancers [11,15,16], and its upregulation is closely associated with poor patient outcomes [11,16]. It has been reported that TGFBI is preferentially expressed in the CSCs of glioblastoma and was upregulated by hypoxia-inducible factor 1 α (HIF1α). TGFBI serves a vital role in maintaining stemlike characteristics by stabilizing the EphA2 protein and stimulating the AKT/c-Myc pathway [17]. Moreover, recent studies have found that TGFBI was markedly increased in muscle-invasive, high-grade, and de novo BLCA and associated with malignant cell transformation and poor prognosis [18–20]. Silencing of TGFBI disrupts the cell cycle of BLCA cells, leading to aberrant G1/S transition and S-phase regulation, as well as impaired cell proliferation and migratory activity [19]. Nevertheless, the mechanisms through which TGFBI regulates cancer stemness and drives tumor progression have not been fully understood.

In the present study, we firstly revealed that TGFBI was upregulated under hypoxic conditions in BLCA cells and played a key role in self-renewal and malignant proliferation. Mechanistically, the TGFBI protein in BLCA cells directly interacts with growth differentiation factor 15 (GDF15) and stabilizes the structural integrity of its disulfide bonds at Cys48 and Cys77, which promotes TGFβ-secreting CAFs’ activation through transforming growth factor beta receptor type 2 (TGFBR2), thereby establishing a TGFBI/GDF15 feedback loop that sustains BLCA stemness. Collectively, we showed that targeting the TGFBI/GDF15 axis and its downstream signaling along with the combined chemotherapy of ponsegromab and cisplatin suppressed tumor metastasis, suggesting an effective therapeutic approach for treatment of metastatic BLCA.

## Results

### The enhanced TGFBI is associated with tumor progression and stemness in BLCA patients

**Fig. 1.**
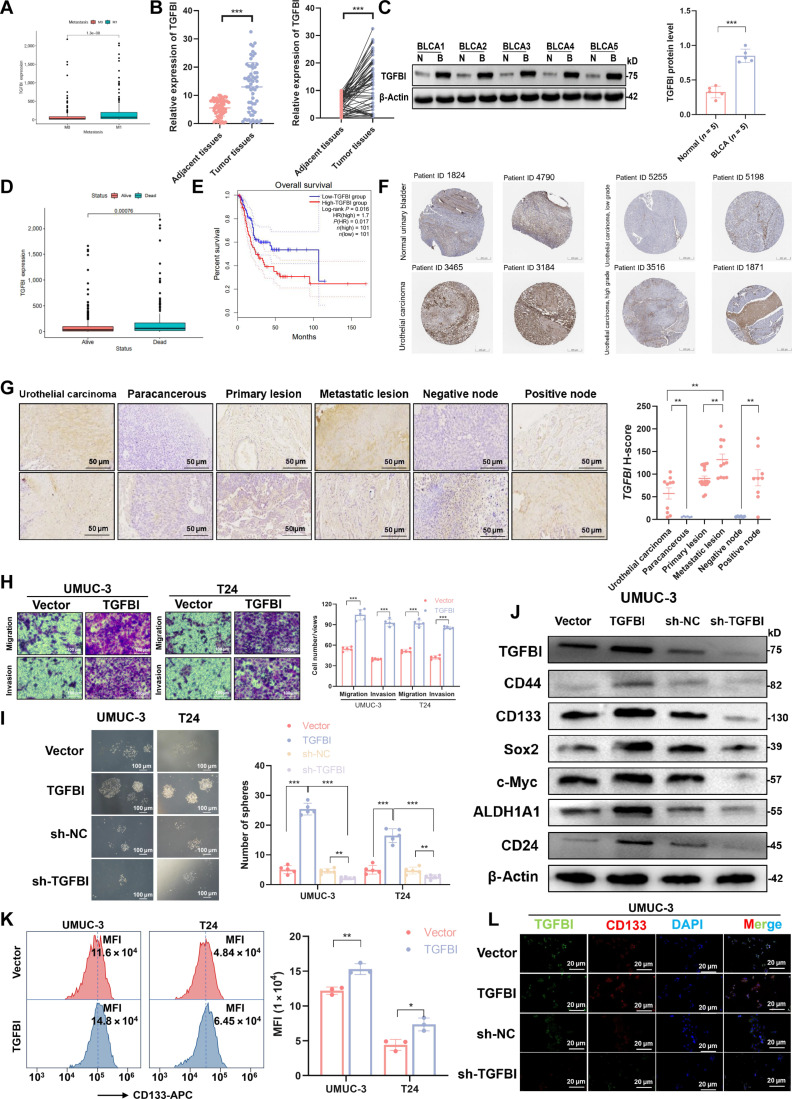
High transforming growth factor beta induced protein (TGFBI) expression enhances cancer stemness, which contributes to unfavorable prognosis in bladder cancer (BLCA). (A) Box diagram reflecting the TGFBI expression of BLCA of different stages and metastases in The Cancer Genome Atlas (TCGA). (B) TGFBI expression in 60 paired collected BLCA tissues and their adjacent normal tissues. (C) A Western blot assay was applied to evaluate the TGFBI protein level in 5 pairs of BLCA tumors (B) and their adjacent normal tissues (N). (D) Box diagram reflecting the TGFBI expression of BLCA of different patients’ statuses in TCGA. (E) The relationship between the overall survival (OS) of BLCA patients and TGFBI expression from the TCGA database. (F) Representative immunohistochemistry (IHC) micrographs of TGFBI in bladder samples and different grades of BLCA from the Human Protein Atlas (HPA) database. (G) Representative IHC images from the BLCA tissue microarray. (H) The Transwell assay showed the enhanced cell migration and invasion abilities of BLCA cells with TGFBI overexpression. (I) A sphere formation assay was conducted to evaluate the stemness properties of BLCA cells after TGFBI overexpression or knockdown. (J) A Western blot assay was applied to determine the expression levels of stemness markers following TGFBI overexpression or knockdown. (K) A flow cytometry assay was performed to determine the CD133 level in BLCA cells following TGFBI overexpression. (L) Immunofluorescence (IF) staining was applied to detect TGFBI and the stem-cell-associated marker CD133 in BLCA cells. Data are presented as mean ± SD. *P < 0.05, **P < 0.01, and ***P < 0.001. HR, hazard ratio; MFI, mean fluorescence intensity.

To explore the role of TGFBI in BLCA, we first assessed TGFBI expression from the BLCA transcriptome sequencing datasets of The Cancer Genome Atlas (TCGA). Compared with that in patients without metastasis, TGFBI expression was significantly upregulated in those with tumor metastasis (Fig. 1A), suggesting its potential prognostic significance. This observation was further validated in an independent BLCA cohort, where TGFBI expression was significantly higher in BLCA samples compared to that in adjacent normal tissues (Fig. 1B and C). Furthermore, the analysis of survival curves in BLCA from TCGA also demonstrated a cause–effect link between increased TGFBI levels and poorer overall survival (Fig. 1D and E). A positive correlation between TGFBI level and degree of tumor progression was also observed in the intratumoral area from the normal urinary bladder tissue and urothelial carcinoma of different grades, as shown by the Human Protein Atlas-database-derived immunohistochemical data (Fig. 1F). To investigate this observation further, we extended our analysis to a human tissue microarray comprising 54 samples from surgically resected BLCA in which we examined TGFBI protein levels. A higher TGFBI histochemistry score (H-score) was obtained in urothelial carcinoma, metastatic lesions, and positive lymph nodes in comparison with that in their paracancerous tissues, primary lesions, and negative lymph nodes (Fig. 1G).

To further evaluate the potential of the TGFBI-mediated invasion of BLCA cells, we selected the UMUC-3 and T24 cell lines as the experimental cell lines and investigated the functional effect of UMUC-3 and T24 cells with overexpressed or silenced TGFBI (Fig. S1A). In BLCA cells with TGFBI overexpression, we observed significant increased colony-formation ability and cell viability, suggesting that the cell proliferation ability was enhanced by TGFBI upregulation (Fig. S1B and C). Our functional assay also confirmed the enhancement of BLCA cells’ migratory and invasive capacities after overexpressing TGFBI, while knockdown of TGFBI decreased motile ability and restrained the scratch closure of BLCA cells, demonstrating the regulation function of TGFBI in malignant BLCA features (Fig. 1H and Fig. S1D). Given the potential role of TGFBI in tumor stemness, we next evaluated the correlation between TGFBI and tumor stemness detected by sphere-forming assays of tumor cells under different TGFBI levels. In contrast to that in vehicle controls, sphere-forming efficiency was significantly increased in BLCA cells with TGFBI overexpression; furthermore, TGFBI knockdown resulted in a reduction in the frequency of sphere formation, suggesting a decrease in stemness in BLCA cells (Fig. 1I). These observations were further confirmed in a set of analysis of stemness-related transcription factors as well as stemness-associated markers, in which the expression levels of stemness-related molecules (c-Myc, ALDH1A1, and SRY-box transcription factor 2 [Sox2]) and urothelial CSC markers (CD133, CD44, and CD24) were increased in BLCA cells with TGFBI overexpression and decreased in BLCA cells after TGFBI knockdown (Fig. 1J and Fig. S1E), as also shown by multiple immunofluorescence (IF) analysis and flow cytometry (Fig. 1K and L and Fig. S1F). Collectively, high TGFBI enhances tumor progression and stemness in BLCA.

### Hypoxia stimulates increased TGFBI expression and function via HIF1A

**Fig. 2.**
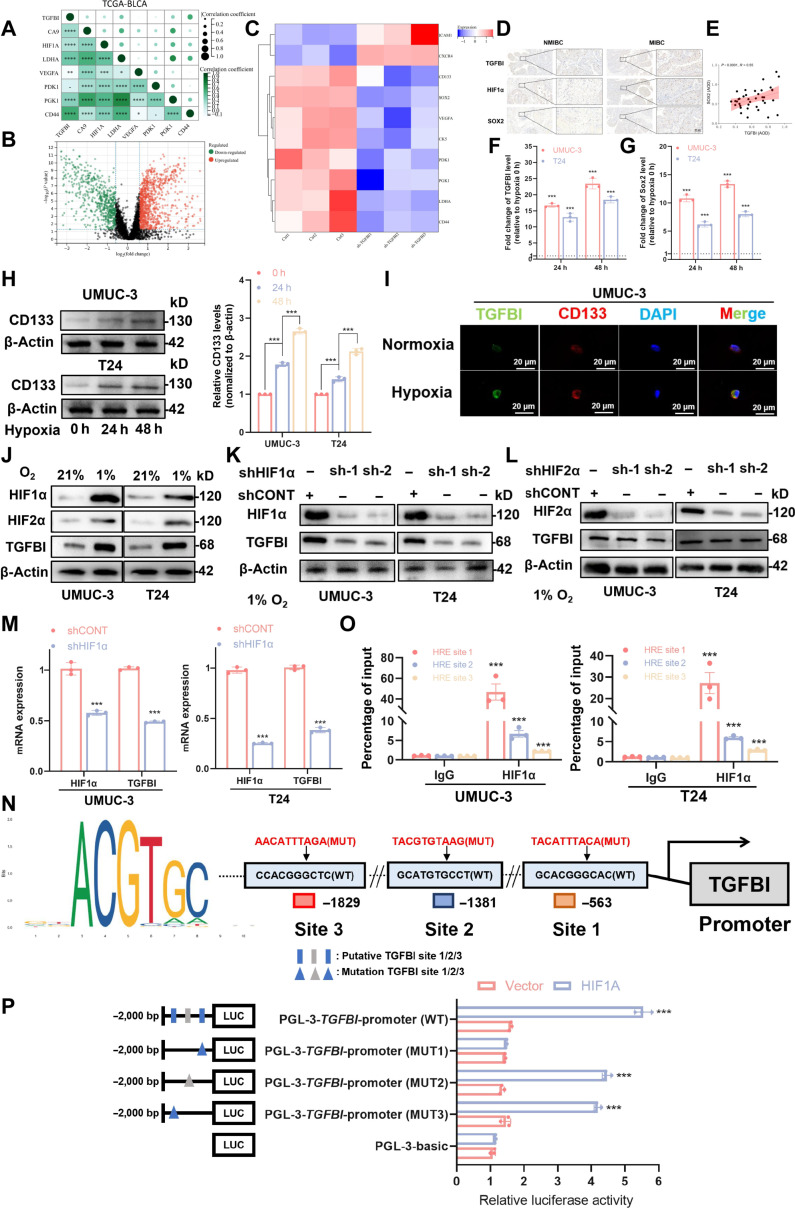
TGFBI levels in BLCA cells are upregulated in a hypoxic microenvironment. (A) TGFBI and hypoxia-related gene expression correlation in the TCGA-BLCA datasets. (B) The results of differentially expressed genes of RNA sequencing (RNA-seq) analysis performed after the knockdown of TGFBI. (C) After TGFBI knockdown, the RNA-seq heatmap shows how the expression of genes linked to hypoxia has changed. (D) TGFBI, hypoxia-inducible factor 1 α (HIF1α), and SRY-box transcription factor 2 (Sox2) IHC staining in various BLCA tissues. (E) IHC staining showing how TGFBI and Sox2 proteins are related in BLCA tissues. (F and G) TGFBI expression in BLCA cells after incubation under normoxia or hypoxia. (H) The amounts of CD133 protein in BLCA cells have grown in either normoxic or hypoxic conditions. (I) TGFBI and stem-cell-associated markers (CD133) stained by IF in BLCA cells in either normoxic or hypoxic conditions. (J) Immunoblotting (IB) was performed to detect TGFBI, HIF1α, and HIF2α in BLCA cells cultured for 24 h under 2 conditions: standard (21% O_2_) and hypoxic (1% O_2_). (K) IB results show TGFBI protein expression in BLCA cells transduced with shCONT or shHIF1α, while those transduced with shHIF2α under hypoxic conditions are presented in (L). (M) TGFBI and HIF1α messenger RNA (mRNA) expression in BLCA cells transduced with shCONT or shHIF1α is demonstrated by real-time quantitative polymerase chain reaction (qRT-PCR) analysis. (N) Schematic diagram displaying 3 predicted HIF1α-binding sites within the TGFBI promoter region and the DNA motif of HIF1A as predicted by JASPAR. (O) Chromatin immunoprecipitation (ChIP) analyses reveal that in hypoxic BLCA cells, HIF1α binds to the TGFBI promoter. (P) Luciferase reporter assays were used to confirm that HIF1A binds to the TGFBI promoter region. The mean ± SD is used to display the data. *P < 0.05, **P < 0.01, ***P < 0.001, and ****P < 0.0001. AOD, average optical density; DAPI, 4′,6-diamidino-2-phenylindole; IgG, immunoglobulin G; HRE, hypoxia response element.

BLCA metastasis and progression are fueled by hypoxia and cytokines in the TME, which increase the stemness of CSCs [21–23]. Several hypoxia markers, such as CA9, HIF1A, PGK1, CD44, PDK1, LDHA, and VEGFA, showed a positive correlation with TGFBI expression, according to our analysis of the TCGA databases (Fig. 2A). Single-sample gene set enrichment analysis (ssGSEA) based on TCGA data also demonstrated that TGFBI was associated with cellular response to hypoxia (Fig. S2A). Subsequently, we explored the correlation between hypoxia and TGFBI. Transcriptome sequencing (RNA sequencing [RNA-seq]) revealed that the knockdown of TGFBI was strongly associated with decreased hypoxia markers (Fig. 2B and C), suggesting TGFBI’s involvement in BLCA development associated with hypoxia pathways. We continue to explore the relationship between hypoxia and TGFBI in BLCA tissue. We found a relationship between Sox2, a crucial transcription factor in BLCA self-renewal, and TGFBI expression in addition to HIF1α (Fig. 2D). A positive linear correlation between the expression levels of TGFBI and Sox2, as well as HIF1A, was revealed by additional immunohistochemistry (IHC) analysis of human BLCA specimens (Fig. 2E).

The molecular mechanisms underlying TGFBI upregulation in BLCA cells as a result of exposure to hypoxic conditions were then investigated. In BLCA cells, hypoxia significantly boosted Sox2 expression and affected TGFBI expression (Fig. 2F and G). CD133, which serves as an important marker for the stemness of BLCA cells [24], was also increased in hypoxia-exposed BLCA cells (Fig. 2H). IF also revealed upregulated TGFBI and CD133 under hypoxic conditions (Fig. 2I and Fig. S2B). Additionally, we confirmed that hypoxic conditions induce BLCA cells to upregulate TGFBI and stemness-associated factor expression (Fig. 2J and Fig. S2C). To verify HIF’s function in TGFBI regulation, HIF1α and HIF2α were knocked down using short hairpin RNA (shRNA). In BLCA cells, silencing of HIF1α, but not HIF2α, resulted in decreased TGFBI expression under hypoxic conditions (Fig. 2K and L). Furthermore, TGFBI messenger RNA expression decreased as a result of HIF1α reduction (Fig. 2M). Based on the JASPAR website, we identified 3 putative HIF1α-binding sites (Fig. 2N). HIF1α’s binding to the TGFBI promoter was further confirmed via chromatin immunoprecipitation (ChIP) analysis (Fig. 2O). The luciferase reporter assay, which was used to further investigate the molecular mechanisms of hypoxia-induced upregulation of TGFBI expression, showed that HIF1A overexpression enhanced luciferase activity in reporter constructs harboring these promoters. This effect was reversed by mutating specific binding sites (Fig. 2P). Collectively, these results underscore the essential role of HIF1α in mediating the predominant expression of TGFBI in hypoxic BLCA cells.

### MFAP4^+^ CAFs are associated with upregulated TGFBI and tumor progression in BLCA

**Fig. 3.**
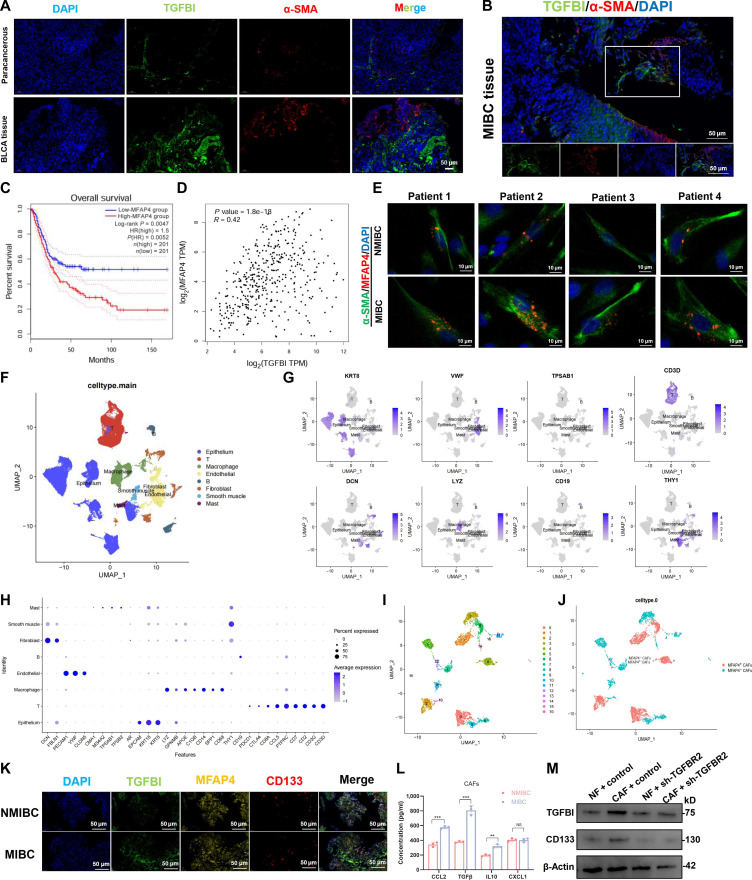
Microfibril-associated glycoprotein 4 (MFAP4)-positive cancer-associated fibroblasts (CAFs) participated in facilitating tumor progression and enhancing cancer cell stemness properties. (A) Double-IF staining showed that TGFBI was highly expressed at the neighboring region of CAFs. (B) Multiplex IF staining verified that TGFBI-expressed BLCA cells in muscle-invasive bladder cancer (MIBC) were next to CAFs (alpha smooth muscle actin [α-SMA] positive). (C) The association between MFAP4 expression from the TCGA database and the OS of BLCA patients. (D) MFAP4 and TGFBI were found to be positively correlated in Gene Expression Profiling Interactive Analysis (GEPIA) data mining. (E) CAFs’ IF staining of MFAP4 and α-SMA. (F) Eight cell clusters that specify the categorization of particular gene signatures are found on a uniform manifold approximation and projection (UMAP) map. (G) Feature plots were used to display the expression patterns of canonical cell-type signatures across each cluster. (H) A point diagram was applied to display marker genes among 8 distinct cell subtypes. (I) Fibroblasts are divided into 17 different subgroups; the UMAP of fibroblasts is annotated by cell type. (J) UMAP plot displaying the distinct CAF subtypes identified based on MFAP4 expression levels. (K) Multiplex IF staining verified that MFAP4-positive CAFs were adjacent to BLCA cells with TGFBI expression. (L) Enzyme-linked immunosorbent assay (ELISA) showed that the levels of TGFβ expressed by the primary CAFs from the MIBC were significantly increased. (M) The expression of TGFBI and cancer stem cell (CSC) markers in transforming growth factor beta receptor type 2 (TGFBR2)-knockdown BLCA cells after co-culture with normal fibroblasts (NFs) and CAFs. The mean ± SD is used to display the data. **P < 0.01 and ***P < 0.001; NS, not significant. NMIBC, non-muscle-invasive bladder cancer. TPM, transcripts per million; KRT8, keratin 8; VWF, von Willebrand factor; TPSAB1, tryptase alpha/beta 1; DCN, decorin; LYZ, lysozyme; THY1, thymocyte antigen 1.

TGFBI is often found to be highly expressed in the adjacent area of CAFs in BLCA, suggesting that CAFs in the microenvironment may be associated with the upregulation of TGFBI (Fig. 3A). The expression pattern of TGFBI in the Comprehensive Repository of Spatial Transcriptomics database (project number: VISDS000132) also suggests a close relationship between CAFs and TGFBI (Fig. S3A). Multiplex immunofluorescence (mIF) staining showed that CAFs were widely distributed around BLCA cells expressing high levels of TGFBI (Fig. 3B). Microfibril-associated glycoprotein 4 (MFAP4) is a critical member of the microfibril-associated protein family in the ECM that is markedly enriched within the tumor stroma of BLCA [25]. Studies have demonstrated that MFAP4 enhances the self-renewal capacity of BLCA cells through the modulation of ECM stiffness to promote the stemness of tumor [26]. ssGSEA based on TCGA data indicated that MFAP4 was involved in tumor progression in BLCA (Fig. S3B). Analysis of BLCA datasets from TCGA indicated that MFAP4 is significantly elevated in patients with tumor progression (Fig. S3C to E). According to survival curves, patients who expressed more MFAP4 had significantly lower chances of surviving (Fig. 3C). Gene Expression Profiling Interactive Analysis (GEPIA)-based BLCA data also revealed that MFAP4 and TGFBI expression are positively correlated (Fig. 3D). We subsequently isolated primary fibroblasts from BLCA tissues. IF confirmed the characteristic markers of CAFs and the upregulation of MFAP4 in MIBC tumor tissues (Fig. 3E). To further elucidate MFAP4’s regulatory role in the TME, we analyzed the Gene Expression Omnibus (GEO) database-derived single-cell dataset (GSE222315). Subsequent analyses comprised 71,975 cells. We performed unbiased clustering across all cells, which was then displayed using uniform manifold approximation and projection (UMAP) for dimension reduction. Cell populations were classified into 8 cell types: keratin 8 (KRT8) and epithelial cell adhesion molecule (EPCAM) were used to identify epithelial cells, von Willebrand factor (VWF) and claudin 5 (CLDN5) were used to identify endothelial cells, decorin (DCN) and fibulin 1 (FBLN1) were used to identify fibroblasts, CD3D and CD3G were used to identify T cells, lysozyme (LYZ) was used to identify macrophages, thymocyte antigen 1 (THY1) was used to identify smooth muscle cells, and cells with high tryptase alpha/beta 1 (TPSAB1) expression, which accounted for a small proportion, were classified as mast cells (Fig. 3F to H). In order to further elucidate the relationship between fibroblasts and BLCA prognosis, fibroblast clusters were extracted separately, re-embedded and reclustered to construct the fibroblast atlas (Fig. 3I). In this atlas, 3,414 fibroblasts were divided into 17 clusters. Subsequent clustering analysis of CAFs based on MFAP4 expression identified distinct MFAP4^+^ and MFAP4^−^ subsets (Fig. 3J). Subsequently, mIF staining of BLCA tissues was conducted. The results showed the upregulation of MFAP4 and TGFBI and their spatial proximity with the cancer stemness marker (Fig. 3K). We examined the secreted factors of CAFs using enzyme-linked immunosorbent assay (ELISA) and found that CAFs isolated from MIBC exhibited a markedly increased secretion of TGFβ (Fig. 3L). Furthermore, co-culture with CAFs upregulated TGFBI and stemness marker expression in BLCA cells compared to those in normal fibroblasts, which can be blocked by TGFBR2 knockdown (Fig. 3M). These results suggest that TGFβ from BLCA-derived CAFs could further induce the expression of TGFBI in cancer cells, thereby establishing a positive feedback loop. Therefore, BLCA cells exhibited extensive interactions with MFAP4^+^ CAFs, and TGFBI could affect BLCA progression through its actions on MFAP4^+^ CAFs.

### TGFBI-promoted tumor-cell-derived GDF15 disulfide bond modification drives tumor progression

**Fig. 4.**
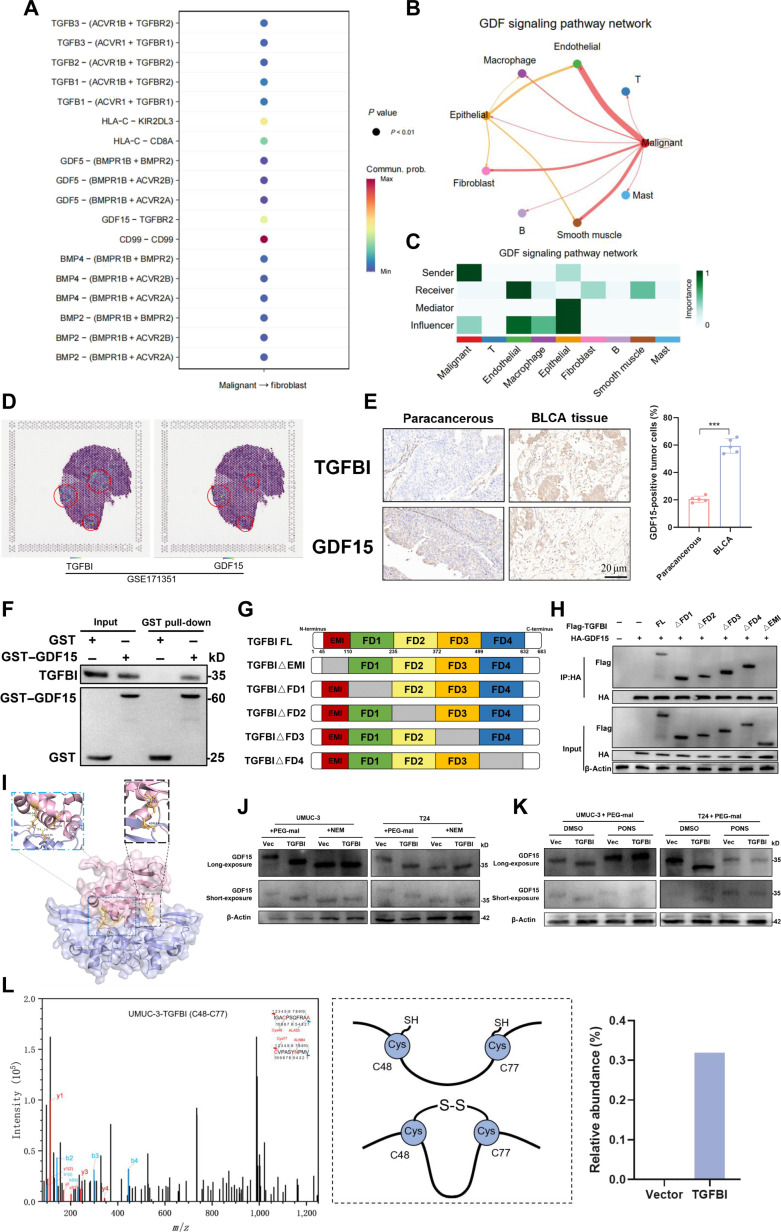
TGFBI promotes the disulfide bond formation of growth differentiation factor 15 (GDF15). (A) Dot plot of interactions among cancer cells and fibroblasts. (B) Inferred interactions of the GDF signaling network between cancer cells and fibroblasts quantified by CellChat. (C) Heatmaps of significant receptor–ligand interactions among cancer cells and fibroblasts using CellphoneDB. (D) Spatial transcriptomics data demonstrate the localization of TGFBI-positive cells and GDF15 expression. (E) IHC staining of TGFBI and GDF15 in different BLCA tissues. (F) Glutathione *S*-transferase (GST) pull-down assays with recombinant purified GST–GDF15 and total proteins from BLCA cells. (G) Schematic diagram of indicated TGFBI truncation constructs. (H) Western blotting was conducted on input and co-immunoprecipitation (co-IP) samples from HEK293T cells transfected with the indicated plasmids. (I) The TGFBI–GDF15 complex was simulated using molecular dynamics, and important interacting residues were visualized structurally. (J) The protein band location of GDF15 from BLCA cells transfected with the vector or TGFBI was displayed by Western blot. (K) As indicated above each blot, protein lysates were subjected to polyethylene glycol maleimide (PEG-mal) subsequent to the exposure of BLCA cells to the GDF15 inhibitor ponsegromab. Western blot assay was applied to examine the location of GDF15 protein bands in BLCA cells transfected with the vector or TGFBI. (L) Mass spectrometry analysis integrated with quantitative bar chart visualization demonstrated the variations in disulfide bond formation between the C48 and C77 residues of GDF15 protein when comparing UMUC-3-Vector cells with UMUC-3-TGFBI cells. The mean ± SD is used to display the data. ***P < 0.001. TGFB3, transforming growth factor beta 3; TGFB2, transforming growth factor beta 2; TGFB1, transforming growth factor beta 1; ACVR1B, activin A receptor type 1B; ACVR1, activin A receptor type 1; ACVR2B, activin A receptor type 2B; ACVR2A, activin A receptor type 2A; HLA-C, human leukocyte antigen-C; KIR2DL3, killer cell immunoglobulin-like receptor 2DL3; BMPR1B, bone morphogenetic protein receptor type 1B; BMPR2, bone morphogenetic protein receptor type 2; BMP4, bone morphogenetic protein 4; BMP2, bone morphogenetic protein 2; HA, hemagglutinin; NEM, *N*-ethylmaleimide; VEC, vector; PONS, ponsegromab; DMSO, dimethyl sulfoxide.

Multiple cues from genetically diverse tumor cells and the TME determine the heterogeneity of CAFs, with tumor cell signals being thought to be the main cue [27–29]. GDF15 signaling was identified as a major player in the investigation of CAFs in BLCA tissues by CellChat and CellphoneDB analyses (Fig. 4A). Figure 4B and C show that GDF15 expression showed robust interactions with its receptors in CAFs, indicating paracrine GDF15 signaling from BLCA cells to CAFs. GDF15 is known to promote tumor malignancy through multiple mechanisms, including regulating cell proliferation, invasion, and angiogenesis. In BLCA spatial transcriptome data, elevated TGFBI and GDF15 expression led us to hypothesize that tumor-cell-derived GDF15 drove tumor stemness and progression (Fig. 4D). We verified the upregulation of TGFBI and GDF15 in BLCA tissues by immunohistochemical staining (Fig. 4E). Subsequently, we overexpressed or silenced GDF15 to explore the role of GDF15 in BLCA. The expression of stemness-associated factors and urothelial CSC markers was increased in BLCA cells with GDF15 overexpression (Fig. S4A). We assessed the functional changes in BLCA cells using wound-healing assays, and the data indicated that GDF15 overexpression promoted tumor proliferation (Fig. S4B). Additionally, the sphere formation assay demonstrated that the frequency of sphere formation was elevated by GDF15 overexpression (Fig. S4C). Collectively, high GDF15 is associated with tumor progression and stemness in BLCA.

Given that both TGFBI and GDF15 belong to the TGFβ-related family, we hypothesize that TGFBI affects the activity of GDF15 through its interaction with GDF15. We then investigated the interaction between TGFBI and GDF15 proteins. Co-immunoprecipitation assays confirmed a strong interaction between TGFBI and GDF15 in BLCA cells (Fig. S4D). Glutathione *S*-transferase (GST) pull-down assay using purified recombinant GST–GDF15 and total proteins isolated from BLCA cells indicated that TGFBI binds directly to GDF15 (Fig. 4F). To identify the specific domains of TGFBI responsible for binding to GDF15, we generated 5 truncation mutants of TGFBI with a FLAG tag (Fig. 4G). Co-immunoprecipitation assays revealed that the EMI domain of TGFBI is essential for its interaction with GDF15 (Fig. 4H). For the purpose of investigating the potential interaction capacity of TGFBI and GDF15, we then conducted molecular docking analysis to verify their binding activity. The complex stability of the TGFBI–GDF15 complex is facilitated by specific amino acid-level interactions, as shown by structural visualization (Fig. 4I). Molecular docking results suggest that the predicted binding sites ALA55 and ALN84 are respectively positioned near the cysteine residues CYS48 and CYS77 of GDF15. Given that GDF15 typically exists as a disulfide-linked dimer [30,31], we hypothesize that TGFBI enhances its activity by promoting the formation of disulfide bonds in GDF15. Protein modification with polyethylene glycol maleimide (PEG-mal) is frequently achieved through the alkylation of free cysteine residues that do not form disulfide bonds [32,33]. The molecular weight of GDF15 in control cells was found to be higher than that in BLCA cells overexpressing TGFBI when PEG-mal was added to the protein lysates of UMUC-3 and T24 cells. This suggests that more disulfide bonds and fewer free cysteine residues were formed following TGFBI overexpression (Fig. 4J). As a control, in vector- or TGFBI-transfected BLCA cells, the molecular weight of GDF15 remained unchanged following treatment with *N*-ethylmaleimide (Fig. 4J). Interestingly, the protein lysates from BLCA cells supplemented with PEG-mal showed an increase in the molecular weight of GDF15 after treatment with the GDF15 inhibitor ponsegromab, which blocked the binding of GDF15 and TGFBI. This suggests that TGFBI promotes the formation of GDF15 disulfide bonds (Fig. 4K). The disulfide bonds of GDF15 were analyzed by mass spectrometry using UMUC-3-Vector and UMUC-3-TGFBI. The findings demonstrated that following TGFBI overexpression, the GDF15 protein enhanced the disulfide binding sites Cys48-Cys77 (Fig. 4L). There results were further confirmed by mutations of C48 and C77, which down-regulated the expression of the CSC marker in UMUC-3 cells transfected with C48 and C77 mutations (Fig. S4E). Therefore, TGFBI promotes disulfide bond modifications of GDF15 and BLCA tumor progression.

### TGFBI enhances the function of CAFs and BLCA cells through the GDF15/TGFBR2 axis by activating the PI3K/AKT signaling pathway

**Fig. 5.**
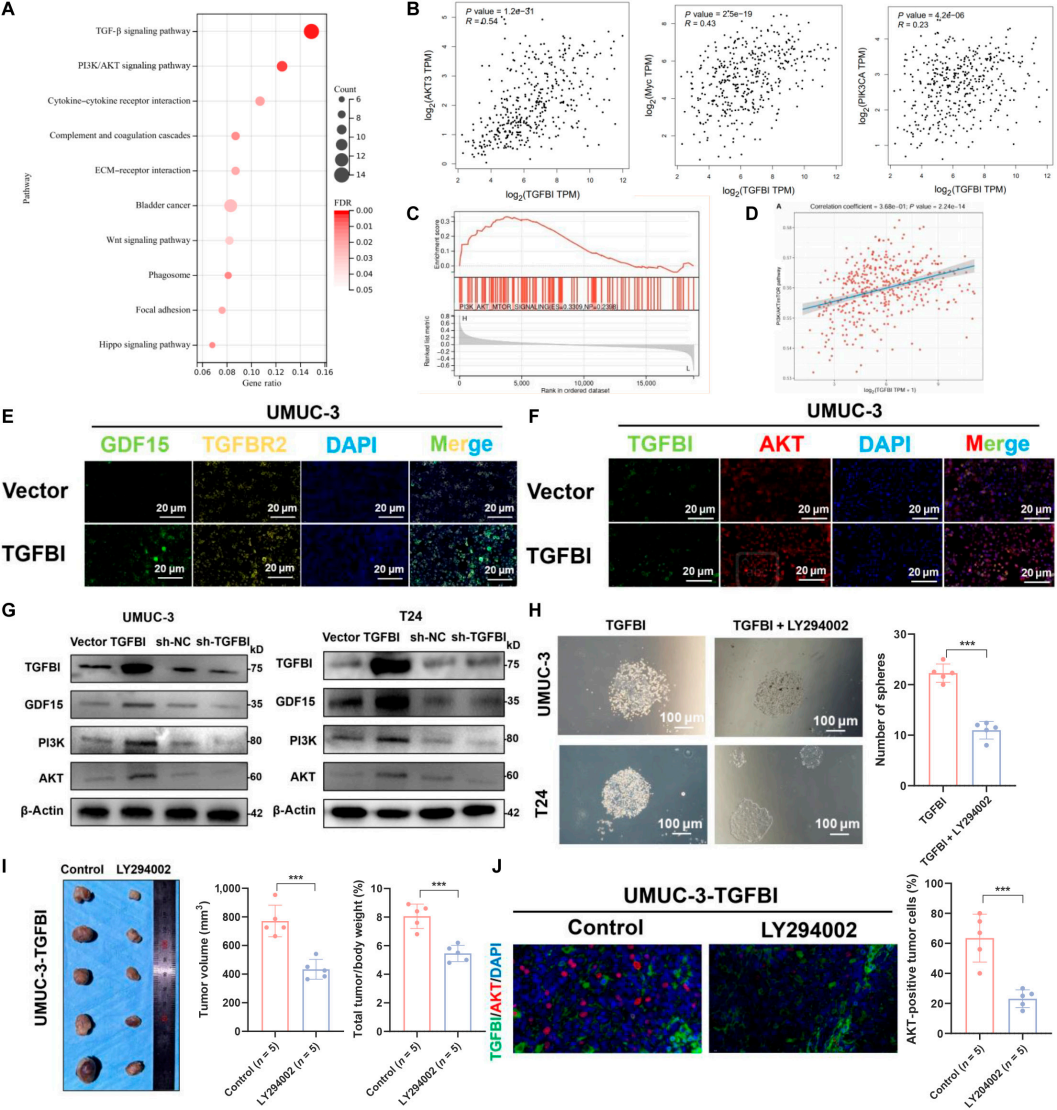
TGFBI exerts its effect on activating the phosphatidylinositol 3-kinase (PI3K)/AKT signaling pathway and enhancing the functions of BLCA cells with the involvement of GDF15 and TGFBR2. (A) Kyoto Encyclopedia of Genes and Genomes (KEGG) enrichment analysis for identified differentially expressed genes (DEGs) in BLCA cells. (B) TGFBI and PI3K/AKT-signaling-pathway-related genes co-expressed in BLCA in the TCGA cohort. (C) Gene set enrichment analysis (GSEA) outcomes demonstrated a positive correlation between the PI3K/AKT/mTOR signaling pathway and TGFBI expression levels. (D) Spearman correlation analysis showing the positive correlation between the PI3K/AKT pathway score and TGFBI expression using the TCGA cohort. (E) IF staining of GDF15 and TGFBR2 in BLCA cells after TGFBI overexpression. (F) IF staining of TGFBI and AKT in BLCA cells after TGFBI overexpression. (G) Total levels of the proteins PI3K and AKT in BLCA cells transfected with various vectors. (H) After TGFBI overexpression and LY294002 treatment, the stemness of BLCA cells was evaluated using the sphere formation assay. (I) LY294002 was applied to xenograft tumors made from UMUC-3 cells, and the weight and volume of the tumors were recorded. (J) Following LY294002 treatment, UMUC-3-TGFBI-derived xenograft tumors underwent double-IF staining using antibodies against TGFBI and AKT. The mean ± SD is used to display the data. ***P < 0.001. ECM, extracellular matrix; FDR, false discovery rate.

For the identification of the downstream targets of the TGFBI–GDF15–TGFBR2 axis, we conducted RNA-seq analysis after silencing TGFBI. Kyoto Encyclopedia of Genes and Genomes analysis of differentially expressed genes revealed BLCA cells highly enriched in the phosphatidylinositol 3-kinase (PI3K)/AKT pathway (Fig. 5A), suggesting that PI3K/AKT played a potential role in tumor progression. we analyzed its correlation with TGFBI using the TCGA database. The analysis revealed that TGFBI expression was significantly associated with PI3K/AKT signaling, including PIKC3A, AKT3, and Myc (Fig. 5B). The PI3K/AKT-signaling-pathway-related genes were enriched in GSEA results (Fig. 5C), which is essential for cancer initiation, development, and angiogenesis and can be activated by GDF15 [34] or TGFBR2 [35]. ssGSEA of TCGA data further indicated that TGFBI was involved in the PI3K/AKT signaling pathway (Fig. 5D). Therefore, we overexpressed or silenced TGFBI in UMUC-3 cells. The results of IF showed that overexpression of TGFBI increased the level of the TGFBR2/PI3K/AKT axis through GDF15 (Fig. 5E and F). The results of Western blot showed that altering TGFBI expression levels affected total PI3K and AKT levels (Fig. 5G). Subsequently, we treated BLCA cells with PI3K/AKT inhibitor LY294002. The sphere formation assay showed that LY294002 decreased the frequency of sphere formation (Fig. 5H). Furthermore, the PI3K/AKT inhibitor LY294002 was used to treat subcutaneous xenograft tumors originating from BLCA cells that overexpress TGFBI. The tumor weight and volume were decreased by the LY294002 treatment in comparison to those of the control group (Fig. 5I). Significantly, LY294002-treated tumors had lower levels of AKT^+^ BLCA cells (Fig. 5J). Overall, these results show that TGFBI activates the PI3K/AKT signaling pathway, which in turn increases tumor stemness.

### Combining ponsegromab and cisplatin represses the growth of the BLCA xenograft model

**Fig. 6.**
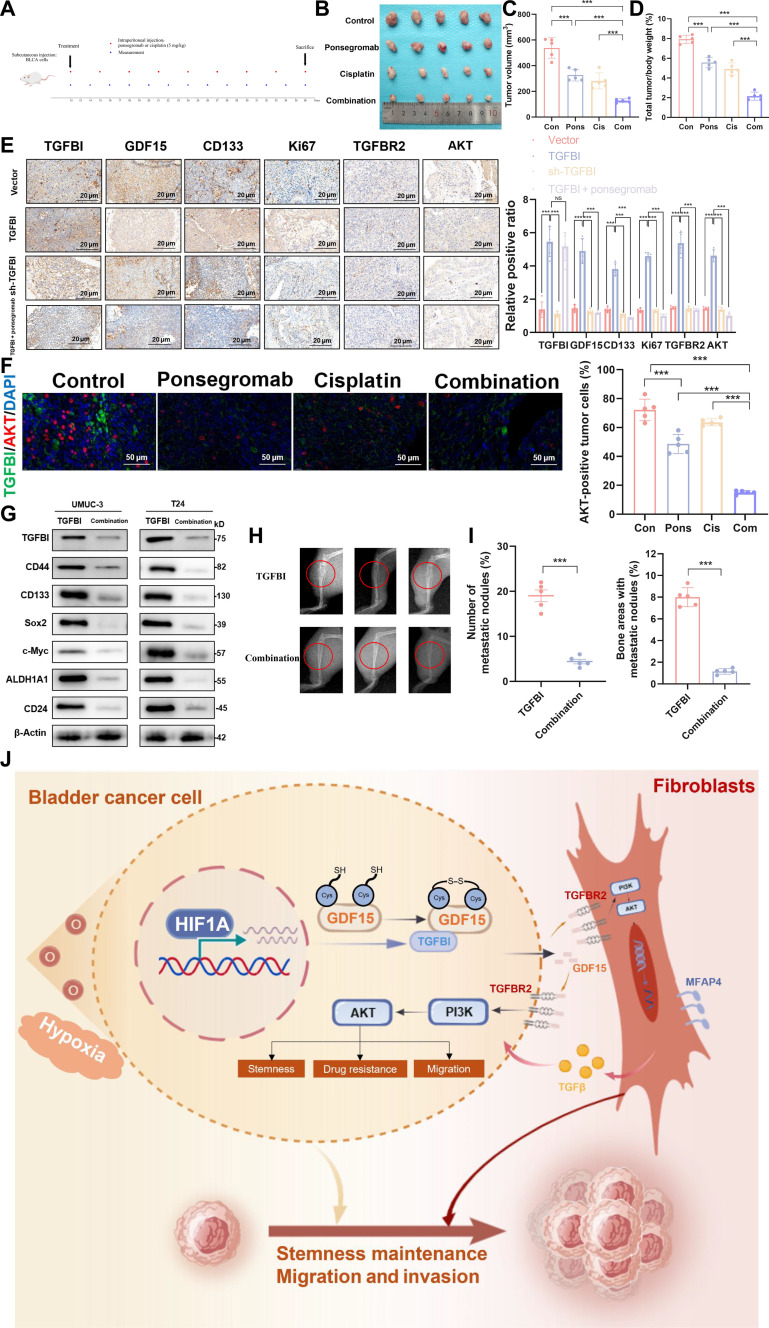
Combining ponsegromab and cisplatin inhibits the tumor progression of the mouse BLCA xenograft model. (A) Diagram showing how each group’s mouse subcutaneous tumor model was set up. (B) Xenograft tumors generated from BLCA cells were administered with ponsegromab (5 mg/kg), cisplatin (5 mg/kg), or their combination, and the tumor volume (C) and weight (D) were determined. (E) Quantification of the indicated protein after IHC staining (scale bar, 50 mm). (F) Double-IF staining was performed to assess the proportion of AKT-positive tumor cells in xenograft tumors subjected to the treatment. (G) Following the recommended treatments, TGFBI and stemness marker protein levels were evaluated using Western blot. (H) Representative images of radiographic image in the tibias of mice. (I) Histologically confirmed bone metastatic lesions (left) and the relative metastatic area in bones (right) of each group. (J) Schematic illustration of the proposed mechanism by which TGFBI induces cancer stemness via the GDF15/TGFBR2/PI3K pathway during BLCA development and progression. The mean ± SD is used to display the data. ***P < 0.001. Con, control; Pons, ponsegromab; Cis, cisplatin; Comb, combination of ponsegromab and cisplatin.

Finally, we explored the potential of blocking TGFBI/GDF15/TGFBR2 axis to suppress BLCA progression in mice. We established a BLCA subcutaneous transplanted tumor model to see if TGFBI enhances tumor stemness and promotes BLCA cell proliferation in vivo through the GDF15/TGFBR2/PI3K pathway. The animal model was established by subcutaneously injecting human BLCA cells into BALB/c-Nude mice. BALB/c-Nude mice received intraperitoneal injections of the GDF15 inhibitor ponsegromab (5 mg/kg) and cisplatin (5 mg/kg) (Fig. 6A). Ponsegromab and cisplatin treatment together dramatically reduced tumor growth in in vivo xenograft tumor experiments (Fig. 6B to D), suggesting that GDF15 inhibition effectively slows the progression of BLCA tumors. IHC was conducted on tumor tissues from the experimental animals to determine the influence of TGFBI and ponsegromab treatment on tumor development in vivo. The findings from IHC analyses indicate that upregulation of TGFBI resulted in increased TGFBI, Ki67, GDF15, and TGFBR2 expression in tumor tissues, while ponsegromab treatment decreased these effects (Fig. 6E). Double-IF staining of xenograft tumors showed a decrease in AKT^+^ tumor cells after combined treatment (Fig. 6F). Western blot also confirmed that the combination treatment decreased the expression of stemness-associated factor and urothelial CSC markers (Fig. 6G). Furthermore, the number of metastatic foci in the bone was reduced in animals treated with ponsegromab compared to that in control mice (Fig. 6H and I).

The in vivo results were similar to those from the in vitro tests. Taken together, the data show that blocking the TGFBI/GDF15/TGFBR2 pathway can reduce human BLCA cell proliferation and migration and increase sensitivity to chemotherapy (Fig. 6J). Therefore, targeting the TGFBI/GDF15/TGFBR2 pathway, in combination with chemotherapy, shows promise in treating BLCA by reducing tumor stemness and promoting chemosensitivity.

## Discussion

BLCA is one of the most common urinary system malignancies, making it an important worldwide health concern. Although BLCA therapy has considerably improved, the prognosis for people with BLCA remains poor [36]. CSCs, like normal stem cells, play a critical function in cell–cell adhesion, cancer stemness maintenance, and treatment resistance. It has been reported [37] that the development of a CSC profile confers partial resistance to T-cell-mediated cytotoxicity. This might be associated with immunotherapy resistance and correlates with reduced surface expression of the CD103 ligand E-cadherin and human leukocyte antigen-A2 (HLA-A2)–neoepitope complexes [37]. SOX9^+^ CSCs in breast cancer upregulate the lipid transporter ATP-binding cassette subfamily A member 12 (ABCA12). ABCA12 supports cancer stemness and chemoresistance via reducing intracellular ceramide levels. Ceramide reduces cancer stemness by blocking the YAP–SOX9 signaling pathway in CSCs. ABCA12 down-regulation lowers cancer stemness and chemoresistance [38]. Furthermore, multiple tumor-bearing mouse models suggest that CSCs are responsible for tumor origin, progression, metastasis, and treatment resistance [39,40]. As a result, there is an urgent need to identify novel therapeutic targets and the regulatory mechanism that governs BLCA cell stemness.

In this study, we comprehensively and thoroughly evaluated the functional impact of TGFBI on cancer stemness properties through integrated approaches including sphere-forming assays and stemness marker profiling, indicating that TGFBI was positively associated with cancer stemness and tumor progression. Together with single-cell RNA sequencing (scRNA-seq) and mIF, this study found that TGFBI was upregulated in BLCA, which may activate the GDF15/TGFBR2/PI3K pathway and cause cancer stemness. Crucially, our research revealed a hitherto unknown mechanism by which TGFBI stabilizes the formation of disulfide bonds in GDF15, thereby increasing its activity. The secreted GDF15 can bind to TGFBR2 on MFAP4^+^ CAFs and BLCA cells, thereby activating the downstream PI3K/AKT signaling pathway. Interestingly, a positive feedback loop is created when TGFβ, which is secreted by the activated CAFs, further encourages the expression of TGFBI. GDF15 inhibition reduced the rate of cancer growth and improved sensitivity to cisplatin, indicating the potential of the TGFBI/GDF15 pathway as a target for BLCA therapy.

Cholangiocarcinoma, renal cancer, pancreatic cancer, and colorectal cancer are among the many cancers for which TGFBI has been thoroughly investigated [11,14,41]. Although it is mainly known as an ECM protein that plays a crucial role in the invasive features of highly malignant tumors, its function in promoting stem cell proliferation has recently drawn a lot of attention [42,43]. The role of TGFBI in BLCA has been further explored in our current work. TGFBI expression in BLCA was found to be low in normoxic conditions and to increase in hypoxic conditions. A worse prognosis for patients is known to be associated with the hypoxic microenvironment, which is known to increase the tumorigenic and self-renewal properties of BLCA [44–46]. The BLCA specimen showed consistent preferential expression of TGFBI in tumor cells located in hypoxic regions, indicating that TGFBI expression was induced by hypoxia. Based on these findings, TGFBI might use new methods to control BLCA in hypoxic environments. Our mechanistic analysis demonstrated that HIF1α binds to the particular binding motif in the TGFBI promoter in BLCA cells when hypoxic. As a key molecule mediating ECM remodeling and tumor invasion, the biological function of TGFBI is highly consistent with the acute hypoxic stress response mediated by HIF1α, which further supports the specific regulatory relationship between HIF1α and TGFBI. The PI3K/AKT signaling pathway is subsequently activated downstream as a result of the elevated TGFBI level binding to GDF15. TGFBI, meanwhile, stabilizes GDF15’s disulfide bond formation. Through the TGFBI/GDF15 and PI3K/AKT axes, TGFBI knockdown significantly decreased the ability of BLCA cells to self-renew and proliferate.

GDF15, a member of the TGFβ superfamily, has been shown to play a pivotal role in blocking the immune system in the TME [47], indicating its important function in tumor progression [48,49]. Research studies have indicated that inhibiting GDF15 sensitizes tumor cells to chemotherapy [50] and suppresses stem cell characteristics [51], which aligns with our findings.

Another finding in this study was that TGFBI binds to 2 disulfide bond sites on GDF15 to enhance its activity and subsequently activated the TGFBR2/PI3K/AKT pathway in both BLCA cells and CAFs. Protein folding and functional maintenance have been found to be significantly impacted by disulfide bond formation, a significant posttranslational modification of proteins [52,53]. Additionally, disulfide bond alterations are essential for the development and spread of tumors. For instance, it has been demonstrated that inhibiting the activity of protein disulfide isomerase, which is highly expressed in a variety of tumors, inhibits the growth and migration of tumor cells [54]. Controlling the formation and breakage of disulfide bonds may affect protein activity and stability, thereby modulating the survival and proliferation of tumor cells [55]. According to this study, GDF15’s function in BLCA cells is improved by disulfide bond formation, which is facilitated by high TGFBI expression. When GDF15 function is impaired, TGFBR2/PI3K/AKT signaling is inhibited and thus down-regulates tumor stemness and progression. Furthermore, the potential role of the PI3K/AKT pathway in BLCA stemness has been increasingly elucidated in recent years [56,57], and our study complements the stemness-related regulatory effects of the TGFβ family on the PI3K/AKT pathway in BLCA.

By using scRNA-seq and bioinformatics analysis, we first demonstrated the regulatory function of MFAP4^+^ CAFs in modulating BLCA cancer stemness. These findings demonstrated that the formation of BLCA tumor stemness is associated with MFAP4^+^ CAFs. MFAP family genes are predominantly expressed in stromal cells, particularly CAFs. MFAP4 in particular showed a strong positive correlation with CAF activation and cancer stemness, as verified by IHC and mIF. CAF has attracted increased attention over the recent decade owing to its critical involvement in tumor genesis and progression [58]. Activated CAFs may promote tumor development, invasion, and metastasis through a variety of pathways, as well as ECM remodeling [59,60]. In addition to TGFβ, activated CAFs also secrete multiple pro-tumorigenic factors such as interleukin (IL)-6 and C-C motif chemokine ligand 2 (CCL2), which form a synergistic regulatory network with TGFβ to modulate tumor progression. In BLCA, CAFs activate the cyclic GMP-AMP synthase (cGAS)–stimulator of interferon genes (STING) signaling pathway in BLCA cells via the WNT5A paracrine mechanism, increasing their proliferation and invasion [61]. Furthermore, investigations have shown that the inflammatory CAF subtype is uniquely related to BLCA recurrence. Moreover, inflammatory CAFs in TME cell–cell cross talk during recurrent BLCA could maintain tumor stemness and epithelial mesenchymal transition through the CD44 receptor of rat cardiac stem cell line-M [62]. Tumor stemness is critical for tumor development. However, it is unknown how activated fibroblasts in the BLCA microenvironment regulate tumor stemness. In this study, we observed that stromal CAFs are closely connected to tumor stemness in bladder tumors. Activated fibroblasts may boost TGFβ synthesis and enhance cancer stemness in BLCA cells via the TGFBR2/PI3K/AKT pathway, hence promoting bladder tumor cell growth and metastasis in vivo. Overall, our findings shed new light on the role of BLCA CAFs in promoting stemness. The main limitation of this study lies in the limited clinical sample size. Future clinically related studies with larger samples and preclinical pharmacokinetic studies on drug application will further improve the feasibility of combination treatment application.

In summary, we demonstrated that HIF1α upregulates TGFBI expression in BLCA under hypoxic conditions. High TGFBI further promotes disulfide bond formation in GDF15, which leads to the activation of the TGFBR2/PI3K/AKT pathway in BLCA cells and CAFs. TGFβ secreted by activated CAFs further promotes the function of TGFBI. BLCA stemness, drug resistance, and metastasis are all improved by this positive feedback loop. The GDF15 inhibitor ponsegromab and chemotherapy together significantly suppress tumor growth and decrease tumor stemness. These results point to a possible treatment plan for BLCA patients who express a lot of TGFBI.

## Materials and Methods

### Clinical samples and cell lines

Chongqing Medical University’s First Affiliated Hospital provided the primary BLCA tissues and the normal bladder tissues that were adjacent for the purposes of Western blot and immunostaining (Chongqing, China). The Committees for Ethical Review at Chongqing Medical University’s First Affiliated Hospital (Chongqing, China) approved the clinical sample using in this study (no. CMUFH-2024-401-01). The 293FT human embryonic kidney cell and 5 BLCA cell lines (5637, T24, RT4, J82, and UMUC-3) were acquired from Beyotime (Nanjing, China). T24 cells were cultured in RPMI 1640 medium, and UM-UC-3 cells in Dulbecco’s modified Eagle medium (DMEM), with both media containing 10% fetal bovine serum (FBS; Sigma-Aldrich, no. 12103C). Each cell was incubated under a temperature of 37 °C and 5% CO_2_.

### Plasmids and transfection

Overexpression or shRNA plasmids of TGFBI, GDF15, HIF1α, and HIF2α were purchased from Servicebio (no. GC1050). The sequences of shRNAs that targeted TGFBI, HIF1α, and HIF2α are provided in Table S1. Transfection Grade Linear Polyethylenimine (PEI MAX) (Polysciences, no. 24765-100) was used to transfect lentiviral transfer plasmids into 293T cells for the production of viruses. A 0.45-μm filter (Beyotime, no. FF345) was used to filter the virus-containing supernatant after it had been collected for 48 to 72 h. The collected virus-containing supernatants were used to infect the BLCA cells. The cells treated with purinomycin were screened to obtain stable cell lines with gene overexpression.

### GEO data analysis and TCGA bioinformatics analysis

Data from the GEO database (GSE222315, SCR_005012) were acquired, and we investigated the differences in gene expression in MFAP4^+^ CAFs. The scRNA data were analyzed using the R package Seurat (v4.0.2). The SC Transform technique was employed to normalize the data. The Harmony method (v0.1.0) was used for eliminating sample batch variation and merging multiple Seurat objects into one dataset. To minimize the number of dimensions in the integrated data, the principal component analysis method was employed. The Find-Neighbors method and FindClusters technique were used for grouping cells based on similar characteristics. The data were displayed using UMAP dimension reduction. For further analysis, we filtered individual cells according to criteria like mitochondrial percentages < 10% of the total unique molecular identifier (UMI) count and UMI counts between 3,000 and 40,000. A conversion to log_2_(UMI + 1) was made from the standardized gene expression scale (UMI). We obtained clinically annotated data from TCGA (SCR_003193) and focused on TGFBI, MFAP4, and GDF15 expressions in patients with BLCA. All bioinformatics functional analyses were carried out using the R software (version 4.2.1).

### Animal experiments

We purchased BALB/c nu/nu mice (Strain No. D000521) from GemPharmatech (Nanjing, China). The obtained mice were housed at Chongqing Medical University’s Experimental Animal Center. Every animal study was carried out in compliance with guidelines that had been authorized by the Institutional Animal Ethics Committee of Chongqing Medical University. BLCA cells were injected into the subcutaneous tissue in order to create a subcutaneous xenogeneic tumor graft model in mice. The tumor volume was calculated as 0.5 × length × width^2^. In the drug treatment experiments, after tumor growth, the mice were divided randomly into control and treatment groups. The control group got vehicle injections every 2 d, whereas the treatment groups received injections of LY294002 (5 mg kg^−1^, Beyotime, no. S1737), ponsegromab (5 mg kg^−1^, Beyotime, no. SIM0059), and cisplatin (5 mg kg^−1^, Beyotime, no. S1552) either individually or combined. The tumors were removed for IF and histological staining at the end of the experiment. Anesthetized 6-week-old nude mice were given injections of BLCA cells (1 × 10^5^) in 10 μl of sterile phosphate-buffered saline (PBS) into their tibia. Skeletal metastasis was carried out in accordance with Campbell et al. [63].

### RNA extraction and qRT-PCR analysis

Total RNA isolation from cultivated cell lines was carried out using TRIzol reagent (Invitrogen, no. 15596018CN) according to the manufacturer’s specifications and translated into complementary DNA with the use of the PrimeScript RT Reagent Kit containing gDNA Eraser (BaoRui Medical Biotechnology Co., Ltd, Beijing, China). Real-time quantitative polymerase chain reaction (qRT-PCR) was conducted with the application of TB Green Premix Ex Taq (Tli RNase H Plus) (Takara Bio (Beijing) Co. Ltd.) and gene-specific primers at 0.3 nM. The sequences of the used primers are as follows: 5′-CTTCGCCCCTAGCAACGAG-3′ (TGFBI F), 5′-CATGTACGTTGCTATCCAGGC-3′ (β-actin F), and 5′-AGTCTAGAGATGCAGCAAGATCTC-3′ (HIF1A F); reverse: 5′-TGAGGGTCATGCCGTGTTTC-3′ (TGFBI R), 5′-CTCCTTAATGTCACGCACGAT-3′ (β-actin R), and 5′-TTCCTCATGGTCACATGGATGAGT-3′ (HIF1A R).

### Western blotting

The protein was extracted by means of radioimmunoprecipitation assay lysis buffer from BLCA cells and tumor tissues and then separated using either 6% or 10% sodium dodecyl sulfate–polyacrylamide gel electrophoresis (SDS-PAGE). The protein was then electrotransferred to a polyvinylidene fluoride membrane. Following a 1-h room-temperature blocking with 5% bovine serum albumin, the membrane was incubated with the primary antibody for an entire night at 4 °C. The membrane was then incubated for 1 h at room temperature with the secondary antibody after being cleaned 3 times with Tris-buffered saline with Tween 20 (TBST). Lastly, TBST was used 3 more times to wash the membrane. Table S2 (Supplementary Materials) lists the primary antibodies along with their dilutions. An ultrasensitive enhanced chemiluminescence detection reagent (Meilunbio, MA0186-2) was used to create the immunoreactive bands.

### Flow cytometry

TGFBI-knockdown or overexpressed BLCA cells were inoculated into 6-well plates. After being harvested and cultured for 24 h, the cells were flushed using cold PBS and stained for 30 min with allophycocyanin-conjugated anti-CD133 (Beyotime, no. AC0137). The cells were then subjected to another cold PBS wash before being subjected to flow cytometry analysis (Beckman CytoFLEX LX).

### IF and IHC

Tumor specimens and cells embedded in optical coherence tomography compound were treated for permeabilization in PBS with 0.5% Triton X-100 (Solarbio, no. T8200) for 20 min after being fixed in 4% paraformaldehyde for 30 min for IF analysis. After blocking with 10% donkey serum at room temperature for 1 h, the samples were incubated with primary antibodies overnight at 4 °C. Samples were then kept at room temperature for an hour to incubate with the corresponding secondary antibodies. Invitrogen’s 4′,6-diamidino-2-phenylindole was used for nuclear staining. An Olympus FV1000 laser confocal microscope was used to take pictures, and the ImageJ software was used for analysis.

For IHC staining, IHC staining was carried out on slices that were 5 mm thick and preserved in paraffin. The slides were deparaffinized, rehydrated, antigen retrieved, and incubated with primary and secondary antibodies before being stained with a 3,3′-diaminobenzidine kit (Servicebio, no. P0202) and counterstained using hematoxylin solution (Servicebio, no. C0105S). The H-score was employed as a semiquantitative measure for TGFBI, HIF1α, and Sox2 protein expression in tumors. In IHC, the histological grading system known as the H-score was employed. Semiquantitative tissue staining was accomplished by numerically representing the number of positive cells and the staining intensity per section. For H-scores ranging from 0 to 300, more data suggest a larger overall positive rate.

### Sphere formation assay

The culture medium used for sphere formation contained 1× DMEM/F12 medium (Gibco, no. 11320033), 0.5% methylcellulose (Beyotime, no. ST1510), 1× B27 supplement (Gibco, no. 17504044), 20 ng ml^−1^ epidermal growth factor (PeproTech, no. GB310039), 10 ng ml^−1^ basic fibroblast growth factor (PeproTech, no. RFGFB50), and 100 U/ml penicillin–streptomycin solution (Servicebio, no. G4016). After cell counting, 1 × 10^4^ tumor cells per milliliter of the prepared culture medium were gently plated into each well of an ultralow-attachment 6-well plate (Servicebio, no. CCP-6N). The plate was incubated in a 37 °C cell incubator containing 5% CO_2_, and following 3 d of culture, images were acquired via a light microscope. The number of spheres was quantified to evaluate the stemness of the tumor cells.

### Cell migration and invasion assays

Cell migration and invasion assays were performed using 8-μm-pore-size Transwell chambers with or without a Matrigel membrane (Servicebio, no. G4131) to measure cell motility. One hour before the assay, the 1:8 DMEM-diluted Matrigel mixture (50 μl) was precoated on the Matrigel membrane at 37 °C. After 24 h of serum starvation, 200 μl of serum-free DMEM was used to seed BLCA cells into the upper chamber (2 × 10^4^), and the lower chamber was supplemented with 500 μl of medium containing 10% FBS. When BLCA cells were cultured for 48 h, their capacity for invasion or migration was evaluated. After wiping away cells from the upper side of the filter with a cotton swab, adherent invasive or migrating cells on the lower membrane surface were fixed with 4% paraformaldehyde (15 min), stained with 0.1% crystal violet (15 min), and counted in 5 randomly selected microscopic fields.

### Primary fibroblasts’ isolation

Fresh BLCA and nearby normal tissues were obtained from patients who underwent radical cystectomy and placed on ice promptly for the isolation of primary fibroblasts. Tissues were digested for 1 h at a temperature of 37 °C using 1 mg/ml collagenase A (Solarbio, no. C8140), and residual blood, connective tissue, and vessels were carefully removed. The enzymatic digestion was stopped, and the mixture was centrifuged after being strained using 70-μm cell strainers. Then, cells were cultured for 3 d at a temperature of 37 °C with 10% FBS-supplemented DMEM/F12 medium in a 5% CO_2_ atmosphere. After discarding nonadherent cells, the remaining cells were subjected to differential trypsinization. Antifibroblast microbeads were added to the reconstituted cell pellet after centrifugation and cell counting (Miltenyi Biotec, no. 130-050-601). In 500 μl of buffer, the mixture was resuspended. After introducing the cell suspension into the column, it was placed in the magnetic field of a suitable MACS separator (Miltenyi Biotec, 130-042-501), and the flow-through that contained the unlabeled cells was thrown away. An appropriate amount of buffer was added dropwise, and after removing the separator, the magnetically labeled cells were eluted. Following isolation, the primary fibroblasts were cultured for additional research. For co-culture of the BLCA cells and fibroblasts, the fibroblasts (1 × 10^6^) were planted into a 6-well plate in growth media for 6 h. After discarding the growth media, 1 ml of FBS-free medium was added to continue the culture for 30 h, and the supernatant was collected as conditioned medium.

### Enzyme-linked immunosorbent assay

Sterile conditioned medium was obtained from cultured CAFs, and ELISA (ELISA LAB, Wuhan) was used following the instructions provided by the manufacturer to measure the levels of CCL2, TGFβ, IL-10, and C-X-C motif chemokine ligand 1 (CXCL1) in CAFs. Every sample test was conducted 3 times. A 450-nm-wavelength enzyme-labeled instrument was used to measure the absorbance (optical density value), and the concentrations of CCL2, TGFβ, IL-10, and CXCL1 in the samples were quantified by means of a standard curve.

### Dual-luciferase assay

To investigate the interaction of HIF1A with the promoter regions of TGFBI, the dual-luciferase reporter gene assay was used for validation; 24-well plates were seeded with HEK293T cells (1 × 10^5^ cells/well). Cotransfection of cells with the PGL3-TGFBI promoter (WT), the mutant PGL3-TGFBI promoter (MUT), pcDNA3.1-HIF1A, and an empty vector was performed. pSV40-Renilla was used as an internal reference. Firefly and *Renilla* luciferase activities were measured using the Dual Luciferase Reporter Assay Kit (Vazyme, China) 48 h later.

### Co-immunoprecipitation

The BLCA cells were first lysed using the protein lysis buffer, which was composed of 50 mM Tris–HCl (pH 7.5), 100 mM NaCl, 1% Triton X-100, 0.1 mM EDTA, 0.5 mM MgCl_2_, and 10% glycerol. After that, antibody-bound beads were added to the cell lysates and incubated for an entire night at 4 °C. Western blotting analysis was performed after the antibody/protein complexes had been boiled with protein loading buffer and rinsed 5 times with lysis buffer.

### GST pull-down assay

The recombinant GST–GDF15 fusion proteins and the control GST proteins were expressed in *Escherichia coli* BL21 cells following isopropyl β-D-1-thiogalactopyranoside induction. For GST pull-down analysis, the fusion proteins were captured by means of glutathione–Sepharose 4B beads (GE Healthcare, Little Chalfont, UK). Approximately 100 μg of the GST–GDF15 fusion protein was incubated with 50 μl of Glutathione Sepharose (Abcam, no. AB193267) at 4 °C for 1 h. Subsequently, protein lysates derived from BLCA cells were introduced into the immobilized GST–GDF15 complexes and maintained at 4 °C overnight. After extensive washing with pre-chilled PBS, the bound fractions were subjected to SDS-PAGE, and the interacting proteins were further verified by Western blotting.

### RNA sequencing

Individual knockdown of TGFBI (*n* = 3) was followed by high-throughput transcriptome sequencing (RNA-seq) of BLCA cells with Illumina technology (USA) to identify genes that were abnormally and specifically expressed. Genes and signaling pathways that were differentially expressed in BLCA cells were found using gene expression profiling and pathway enrichment analyses. Illumina’s standard RNA-seq protocol was followed for both sequencing and analysis.

### ChIP assay

We used EZ-Magna ChIP A/G Chromatin IP Kit (Beyotime, no. 17-10461) for ChIP assays following manufacturer’s recommended guidelines. BLCA cells were cultivated for 12 h in a hypoxic environment before being harvested; 5 μg of rabbit pre-immune immunoglobulin G antibody and anti-HIF1α (Beyotime, no. AF7087) antibody were used for the ChIP reaction. By using qRT-PCR (Applied Biosystems), the relative enrichment of the target genes was examined. The following primers were used for the TGFBI promoter ChIP-PCR assay: site 1, forward, 5′-ACCACCATATTCGGGCTTTG-3′, reverse, 5′-TGAGGAATGCTGCCAACAAG-3′; site 2, forward, 5′-TTCGTGAATGCATGCCTCAG-3′, reverse, 5′-AGCCCAAGCCAATGATGTTC-3′; and site 3, forward, 5′-TTGGCGTTTCTGTGGATCAC-3′, reverse, 5′-TGGCCCATCAGACTTGTTTC-3′.

### Mass spectrometry analysis

Pierce Classic Magnetic IP/Co-Immunoprecipitation Kit (Thermo Scientific, no. 88804) was used to process and obtain proteins before mass spectrometry. In short, BLCA cell lysates were exposed to an IP assay with a TGFBI antibody. Magnetic beads were used to enrich proteins that interacted with TGFBI. Samples were then prepared for mass spectrometry analysis by enzymatic digestion, peptide extraction, desalting, washing, reduction, and alkylation. Proteomic analysis was then performed on the samples with the Nalc 1200-FAIMS Fusion Orbitrap-based mass spectrometer. Additionally, a GDF15 antibody was used in an IP assay on whole-cell protein lysates from target cell lines that overexpressed TGFBI. Following boiling of the resultant protein complexes in loading buffer, Western blot was used to evaluate GDF15’s binding to TGFBI.

### Statistical analysis

GraphPad Prism (v9.0) was employed for graph construction and statistical analyses, with results expressed as mean ± SD. Analysis of variance was used to assess several groups, while the Student *t* test was used to compare 2 groups individually. The correlation between 2 genes’ expression levels was determined using Pearson correlation analysis. A *P* value of <0.05 was considered statistically significant.

## Data Availability

The data generated in the present study are available from the corresponding authors upon reasonable request.
