# Supplementary material for: Hypoxia-Induced TGFBI Promotes Bladder Cancer Progression by Creating a Stemness Regulation Loop through Stabilizing the Disulfide Bonds of GDF15
Source: Research (Wash D C). 2026 Feb 11;9:1134. doi: 10.34133/research.1134 (PMC12891367; doi:10.34133/research.1134)
Supplement: Supplementary 1 — Figs. S1 to S4 Tables S1 and S2 [file research.1134.f1.docx]

**Supporting Information**

**Structural integrity of disulfide bonds stabilized by TGFBI creates a stemness regulation loop in bladder cancer**

**Including:**

- **Supporting Figures and Figure Legends**

Figure S1-S4.

- **Supporting Tables**

Table S1-S2.

| 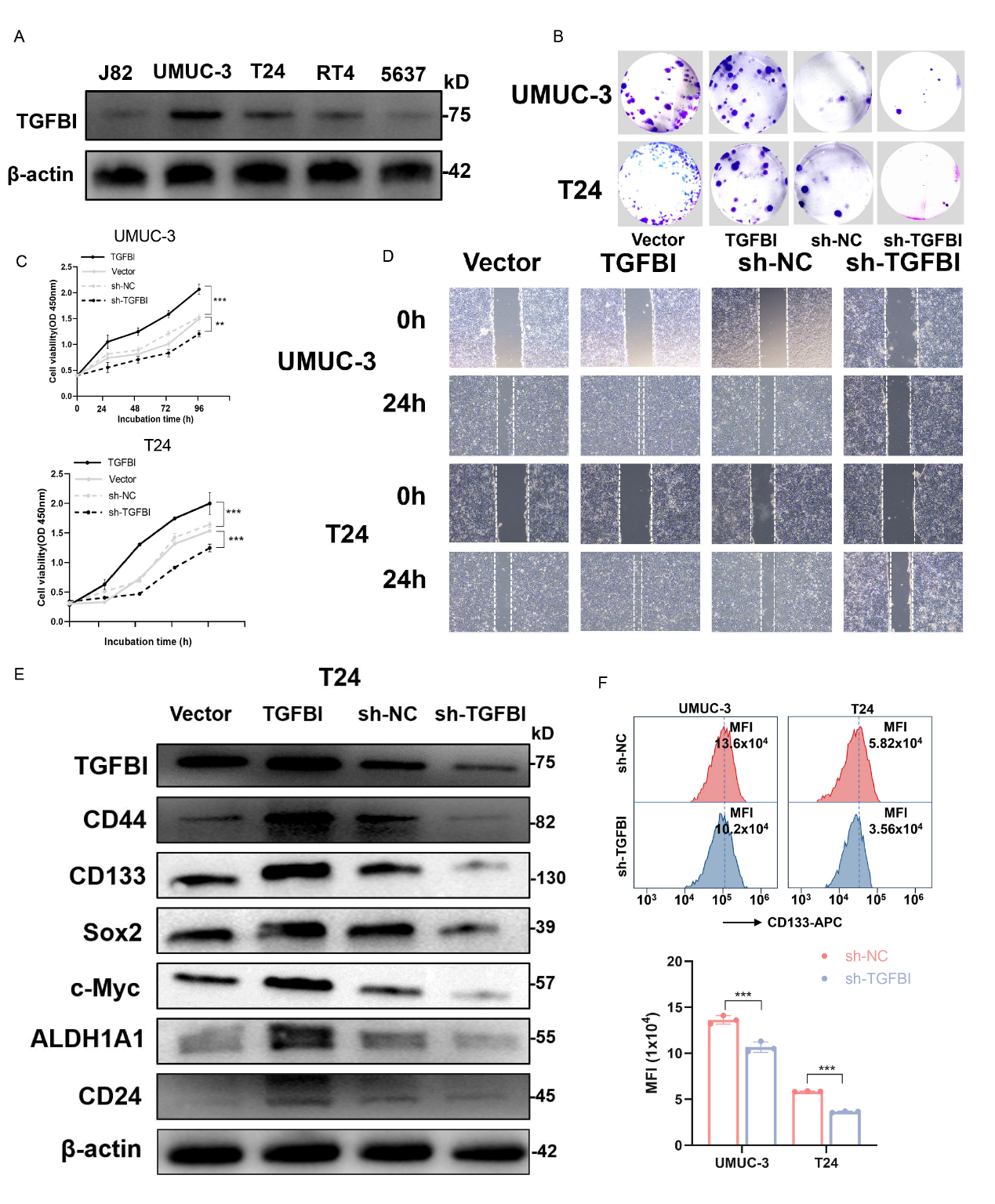 |
| --- |

**Figure S1. TGFBI is overexpressed in BLCA and correlates with tumor progression and stemness.**

**(A)** Western blot was used to assess the protein level of TGFBI in five BLCA cell lines.

**(B)** Functional assay of TGFBI-overexpressing or -knocked down cells detected by plate colony assays **(C),** Cell Counting Kit-8 (CCK-8) assays and by wound healing assays**(D)**.

**(E)** Western blot was used to detect the expression of stemness markers after TGFBI overexpression or knockdown.

**(****F)** Flow Cytometry was conducted to measure the level of CD133 in BLCA cells after TGFBI overexpression.

| 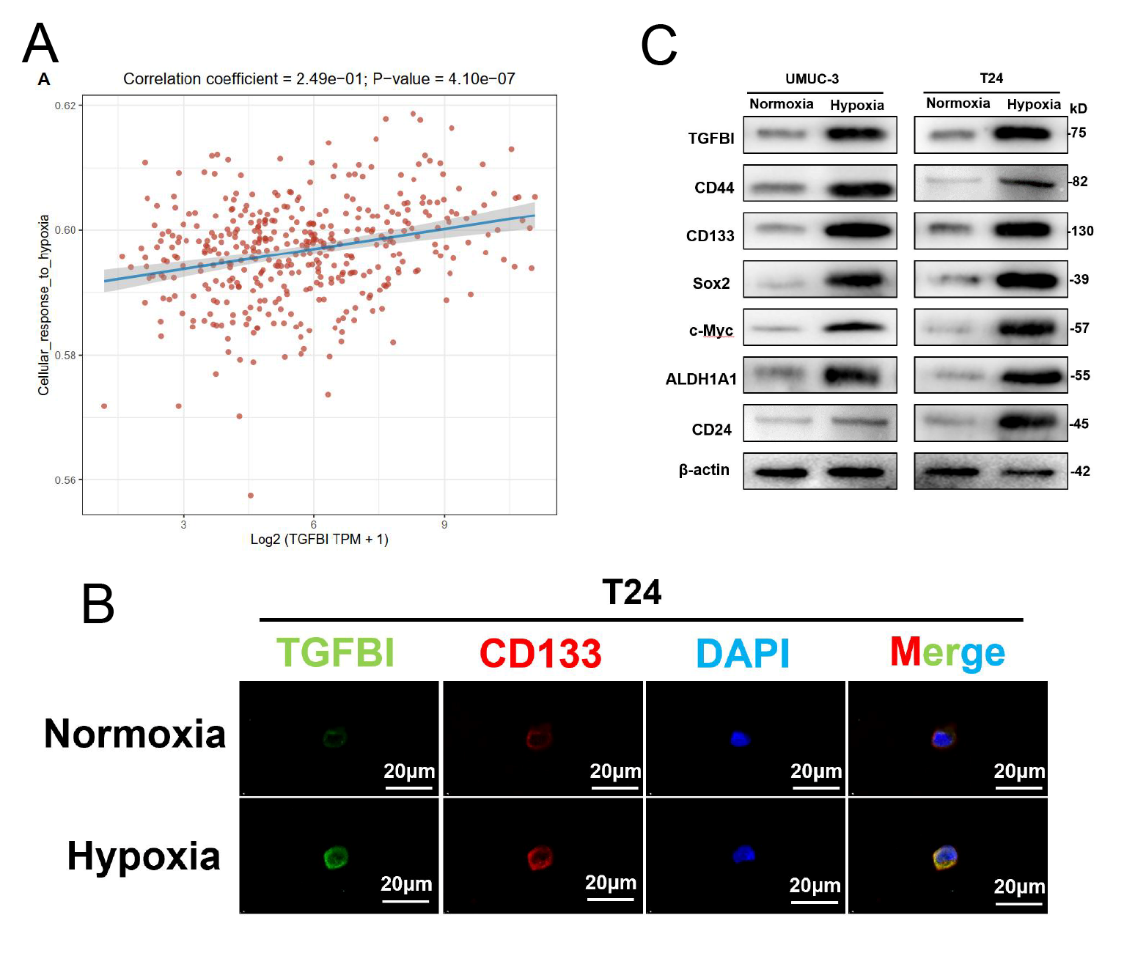 |
| --- |

**Figure S2. TGFBI is induced by HIF1α in BLCA under hypoxia.**

**(A)** Spearman correlation analysis showing the positive correlation between indicated pathway score and TGFBI expression using TCGA cohort.

**(B)** IF staining of TGFBI and stem cell-associated markers (CD133) in BLCA cells under normoxia or hypoxia.

**(C)** Western blot was used to detect the expression of stemness markers in BLCA cells under normoxia or hypoxia.

| 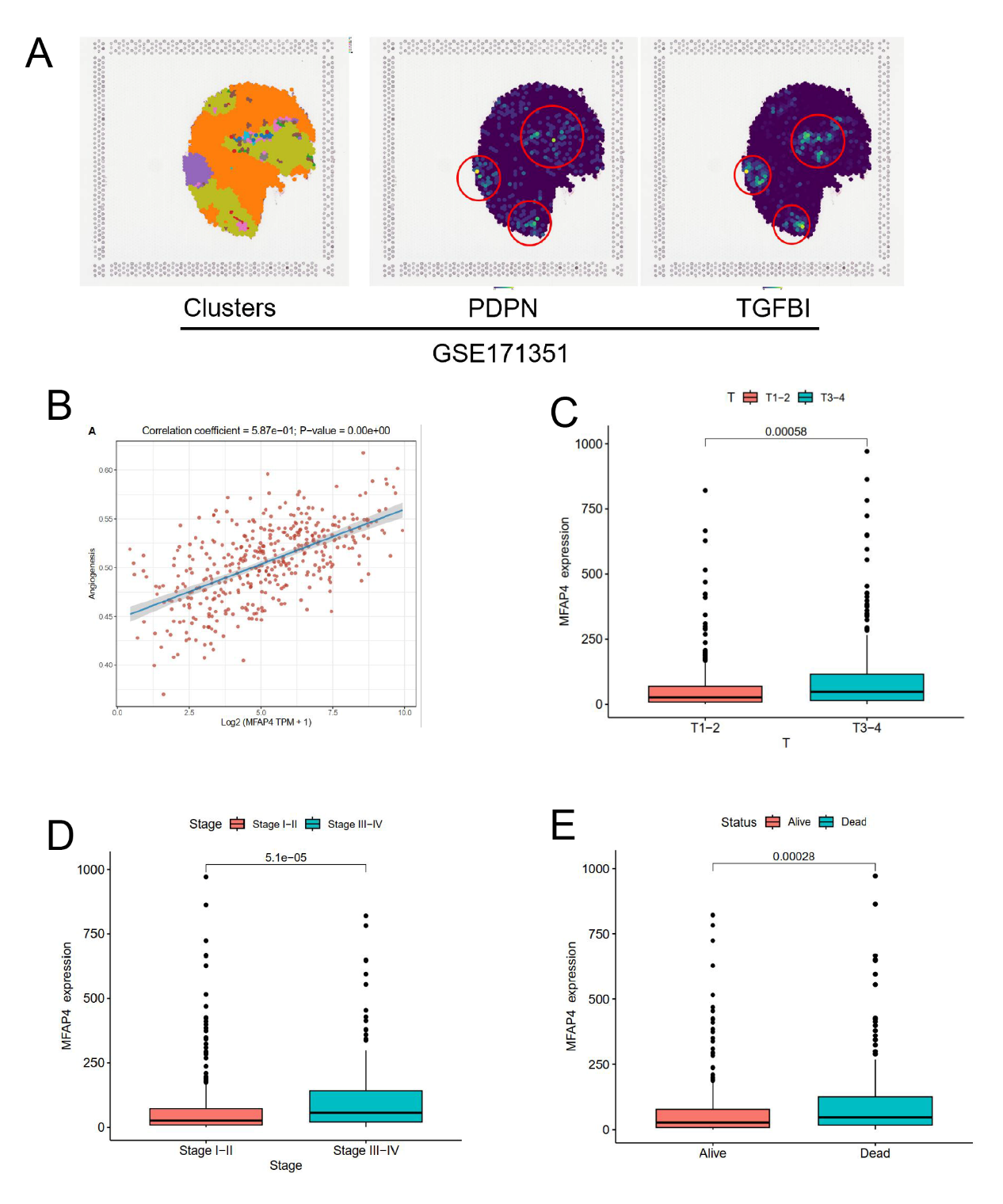 |
| --- |

**Figure S3. MFAP4^+^CAFs were involved in promoting the tumor progression of BLCA.**

**(A)** Spatial transcriptomics data demonstrate the localization of TGFBI-positive cells and the CAF infiltration around these cells.

**(B)** Spearman correlation analysis showing the positive correlation between indicated pathway score and MFAP4 expression using TCGA cohort.

**(CDE)** Box diagram reflecting MFAP4 expression of the indicated cluster.

| 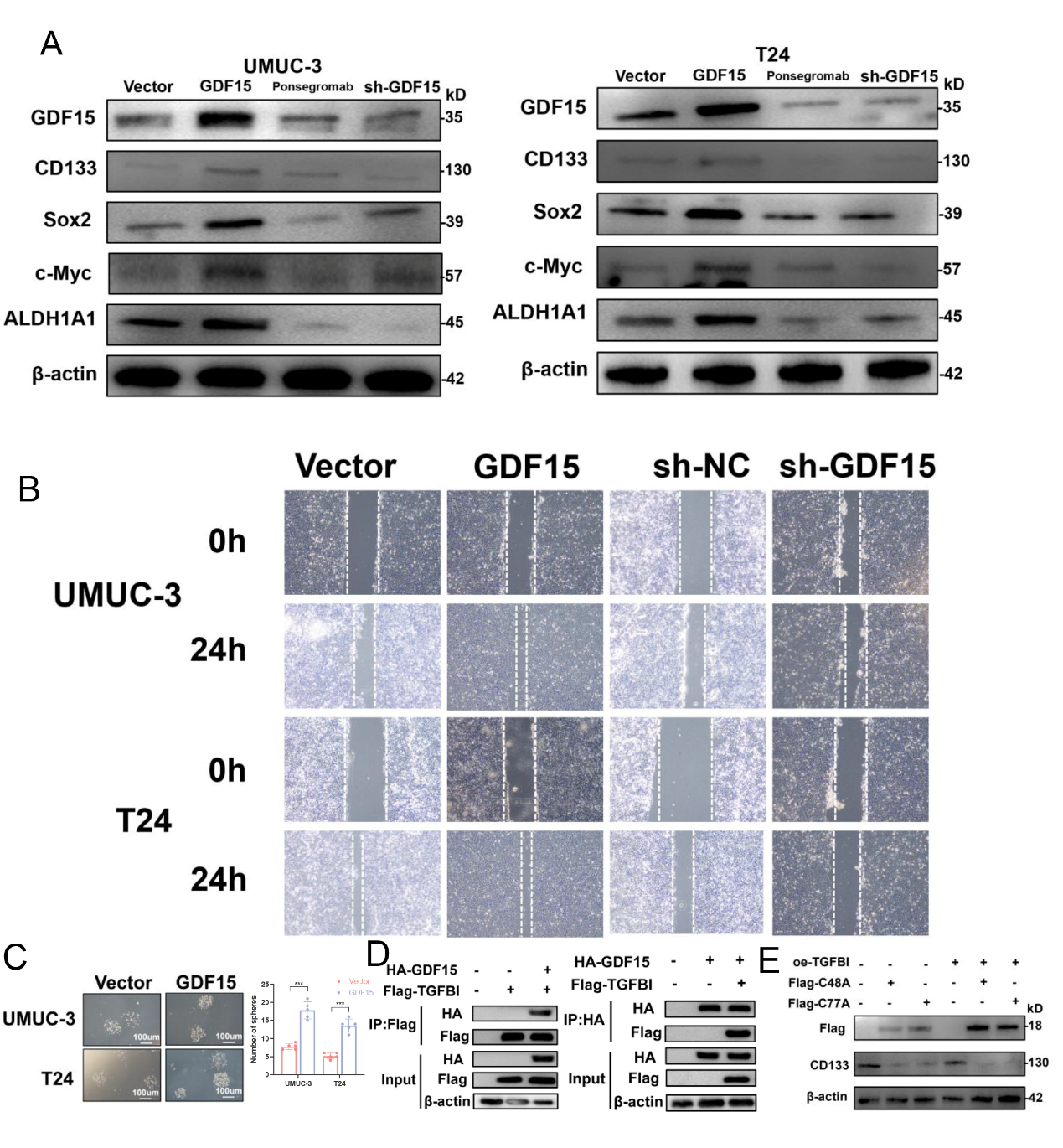 |
| --- |

**Figure S4. TGFBI binds to GDF15 and promote tumor stemness.**

**(A)** Western blot was used to detect the expression of stemness markers after GDF15 overexpression or knockdown.

**(B)** Proliferation of GDF15-overexpressing or -knocked down cells detected by wound healing assays.

**(C)** Sphere formation assay was performed to assess the stemness of BLCA cells following TGFBI overexpression.

**(D)** Co-IP analysis was performed to confirm the interaction between TGFBI and GDF15 in BLCA cells.

(**E**) Expression of CSC markers in BLCA cells after point mutation in C48A and C77A.

**Table S1**

**Sequences used for knocking down**

| **Gene Name** | **Sequences** |
| --- | --- |
| shTGFBI-1 sense | 5′-GCGCUUGAGAUCUUCAAACAATT-3′ |
| shTGFBI-1 antisense | 5′-UUGUUUGAAGAUCUCAAGCGCTT-3′ |
| shHIF1α-1 sense | 5′-CCGCUGGAGACACAAUCAUAUTT-3′ |
| shHIF1α-1 antisense | 5′-AUAUGAUUGUGUCUCCAGCGGTT-3′ |
| shHIF1α-2 sense | 5′-CCUUCGACACAGAUACAUUUUTT-3′ |
| shHIF1α-2 antisense | 5′-AAAAUGUAUCUGUGUCGAAGGTT-3′ |
| shHIF2α-1 sense | 5′-GUUCUGGUGACUCUUGGUCTT-3′ |
| shHIF2α-1 antisense | 5′-GACCAAGAGUCACCAGAACTT-3′ |
| shHIF2α-2 sense | 5′-GCGACAGCUGGAGUAUGAATT-3′ |
| shHIF2α-2 antisense | 5′-UUCAUACUCCAGCUGUCGCTT-3′ |
| shGDF15 sense | 5′-CCGGAUACUCACGCCAGAAGUTT-3′ |
| shGDF15 antisense | 5′-ACUUCUGGCGUGAGUAUCCGGTT-3′ |
| shTGFBR2 sense | 5′-GAAGAAUAUAACACCAGCAAUTT-3′ |
| shTGFBR2 antisense | 5′-AUUGCUGGUGUUAUAUUCUUCTT-3′ |

**Table S2**

**Antibodies required for the western blotting**

| **Antibody Name** | **Dilution** | **Cat No.** |
| --- | --- | --- |
| Anti-TGFBI (Thermo) | 1:1000 | OTI9A11 |
| Anti-HIF1α (Proteintech) | 1:1000 | 20960-1-AP |
| Anti-HIF2α (Servicebio) | 1:1000 | P97481 |
| Anti-c-Myc (Abcam) | 1:100 | ab32072 |
| Anti- ALDH1A1 (Protientech) | 1:500 | 60171-1-Ig |
| Anti- Sox2 (Protientech) | 1:500 | 66411-1-Ig |
| Anti-CD44 (Beyotime, China) | 1:1000 | AF1858 |
| Anti-CD24 (Beyotime, China) | 1:1000 | AF6402 |
| Anti-CD133 (Servicebio, China) | 1:1000 | O5499 |
| Anti-β-actin (Servicebio, China) | 1:2000 | P60710 |
| Anti-GDF15 (Beyotime, China) | 1:1000 | AF6975 |
| Anti-PI3K (Thermo, USA) | 1:2000 | AB_2816587 |
| Anti-AKT (Cell Signaling Technology) | 1:1000 | 9272 |
